# Reproducibility of graph metrics of human brain structural networks

**DOI:** 10.3389/fninf.2014.00046

**Published:** 2014-05-07

**Authors:** Jeffrey T. Duda, Philip A. Cook, James C. Gee

**Affiliations:** Penn Image Computing and Science Laboratory, Department of Radiology, University of PennsylvaniaPhiladelphia, PA, USA

**Keywords:** structure, tractography, connectivity, brain, network, reproducibility, graph

## Abstract

Recent interest in human brain connectivity has led to the application of graph theoretical analysis to human brain structural networks, in particular white matter connectivity inferred from diffusion imaging and fiber tractography. While these methods have been used to study a variety of patient populations, there has been less examination of the reproducibility of these methods. A number of tractography algorithms exist and many of these are known to be sensitive to user-selected parameters. The methods used to derive a connectivity matrix from fiber tractography output may also influence the resulting graph metrics. Here we examine how these algorithm and parameter choices influence the reproducibility of proposed graph metrics on a publicly available test-retest dataset consisting of 21 healthy adults. The dice coefficient is used to examine topological similarity of constant density subgraphs both within and between subjects. Seven graph metrics are examined here: mean clustering coefficient, characteristic path length, largest connected component size, assortativity, global efficiency, local efficiency, and rich club coefficient. The reproducibility of these network summary measures is examined using the intraclass correlation coefficient (ICC). Graph curves are created by treating the graph metrics as functions of a parameter such as graph density. Functional data analysis techniques are used to examine differences in graph measures that result from the choice of fiber tracking algorithm. The graph metrics consistently showed good levels of reproducibility as measured with ICC, with the exception of some instability at low graph density levels. The global and local efficiency measures were the most robust to the choice of fiber tracking algorithm.

## 1. Introduction

Combining magnetic resonance imaging (MRI) of the human brain with graph theory analysis has emerged as a powerful approach to studying large-scale networks of both structural and functional connectivity. In the case of structural connectivity, the use of diffusion weighted MRI and associated white matter fiber tractography methods provide the ability to identify the long-range pathways that connect cortical regions and form a network architecture (Xue et al., 1999; Basser et al., 2000; Hagmann et al., 2003; Lazar et al., 2003). The use of graph theoretical analysis to study the topology and structure of these large scale networks is an increasingly active topic of research (Hagmann et al., 2008; Zalesky et al., 2010; Sporns, 2011; Bastiani et al., 2012; Cheng et al., 2012b; Fornito et al., 2012; Irimia and Van Horn, 2012). These methods have been used to examine the structural consequences of neurological disorders (Guye et al., 2010; Martin, 2012; Xie and He, 2012) as well as the relationship between structure and function (Honey et al., 2007, 2009; Hagmann et al., 2008).

Previous studies examining the reproducibility of graph-based metrics in functional networks have shown good levels of reproducibility in MEG (Deuker et al., 2009), fMRI using BOLD contrast (Telesford et al., 2010; Schwarz and McGonigle, 2011; Braun et al., 2012; Liang et al., 2012; Weber et al., 2013) and arterial spin labeling (Weber et al., 2013). A number of studies have also examined reproducibility in structural networks, each focusing on various aspects of the complex processing pipeline that is a prerequisite for these measures. These have included studies of diffusion spectrum imaging (Bassett et al., 2011; Cammoun et al., 2012) and high angular resolution diffusion imaging (Dennis et al., 2012). Some studies have examined probabilistic tractography (Vaessen et al., 2010; Owen et al., 2013). Diffusion tensor imaging (DTI) based studies using deterministic tractography have included the examination of tractography seed density (Cheng et al., 2012a), anatomic label density (Bassett et al., 2011), and studies examining a variety of network measures (Cheng et al., 2012a; Irimia and Van Horn, 2012). A recent review article discussed the reproducibility of these graph metrics as used to examine both functional and structural networks across a variety of conditions (Telesford et al., 2013).

In this paper we constrain our analysis to DTI-based deterministic fiber tractography. Within this constraint, we examine multiple algorithms for computing streamlines to examine their influence on the final graph metrics. A set of manually defined cortical parcellations (Klein and Tourville, 2012) is used along with a more common template-based parcellation scheme (Tzourio-Mazoyer et al., 2002). The intraclass correlation coefficient (ICC) is used to examine the reproducibility of network summary measures that results from combinations of fiber tracking algorithm and anatomical label set. The dice coefficient provides a measure of topographical similarity to examine the reproducibility of subgraphs extracted as a function of graph density. Graph curves are constructed for a variety of metrics and functional data analysis is used to examine how these metrics differ as a function of graph density or other parameters that are specific to a given metric. We use freely available data and software to create a framework that facilitates future extensions that may examine additional aspects of the processing as well as the comparison to, or addition of, multiple imaging modalities.

## 2. Materials and methods

### 2.1. Neuroimaging data

The Multi-Modal MRI Reproducibility Resource (Landman et al., 2011), informally known as the Kirby dataset (http://www.nitrc.org/projects/multimodal), provides a publicly available test-retest data set consisting of 21 healthy control subjects (11 males). The mean age is 31.76 ± 9.35 with a range of [22, 61]. This data set provides a multitude of MR image types, but here only the T1-weighted anatomical images and diffusion tensor images are examined. The T1 images have a resolution of 1.2 × 1.0 × 1.0 mm. The distributed diffusion images have a resolution of 0.828125 × 0.828125 × 2.2 mm. The diffusion data includes a single *b* = 0 volume and 34 directional diffusion weighted images acquired with *b* = 700 s/mm^2^.

### 2.2. Anatomical labeling

A graph consists of nodes and the edges that connect those nodes. To construct a graph from a brain, a set of anatomical labels are used to define the nodes of the graph. To determine if manually defined cortical labels would provide an inherent advantage in reproducibility we used the Mindboggle dataset which provides a set of manually drawn cortical regions (DKT31) along with a skull-stripped image for a single time point for each subject in the Kirby data set (Klein and Tourville, 2012). To utilize these labels in network creation we performed an intra subject registration between each subject's two T1 images. A brain mask was created from the provided skull-stripped T1 image by thresholding and a morphological closing. This mask was warped into the unlabeled T1 image space and used to create a skull-stripped image. For each time, a transformation was found between the skull-stripped T1 image and the *b* = 0 image, acquired as part of the DTI acquisition. In all subjects, the manually defined labels were propagated into the DTI space for both time points using the appropriate composed transform.

**Figure 1:**
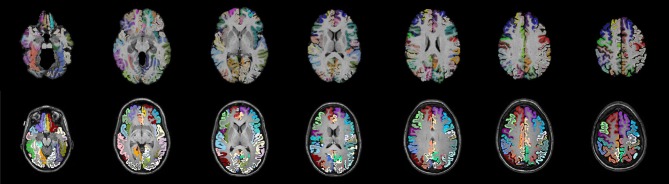
**Two sets of anatomical labels are used to define the networks**. The template based AAL labels (top) and The DKT31 manually defied labels that are provided via Mindboggle (bottom). The AAL labels have been masked to only include gray matter.

**Figure 2:**
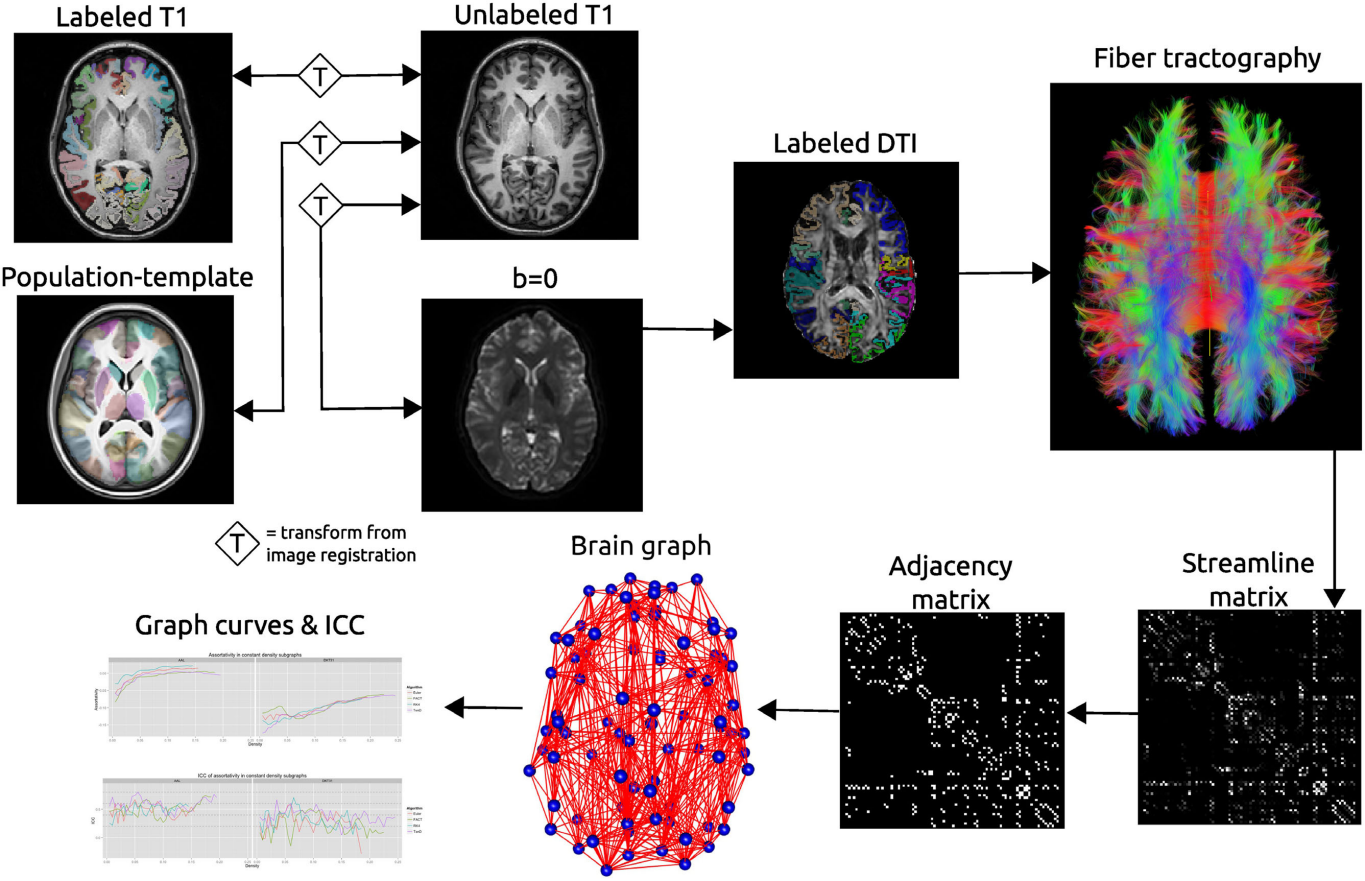
**Schematic of the network processing scheme**. Image registration is used to find transformations between the T1 image and: the T1 image for that subject's other time point; the population template; the *b* = 0 image acquired as part of the DTI acquisition. Labels are transformed into the DTI space where fiber tractography is performed. A matrix is created that records the number of streamline connecting each pair of labeled regions. This matrix is thresholded as constant density to create an adjacency matrix which defines connections in a brain graph. Graph curves are generate by calculating network summary measures over a range of density values and ICC plots are used to examine the reproducibility of the metrics.

One of the most common label sets used in studies of both functional and structural connectivity is the AAL label set (Tzourio-Mazoyer et al., 2002) which is a template based label set. An existing multivariate template had been created from the Kirby dataset using the antsMultivariateTemplateConstruction.sh tool, part of the Advanced Normalization Tools (ANTs) software package (Avants et al., 2009). The antsRegistration tool was used to find a deformable mapping between the T1 template image distributed with the AAL label and the population specific template created from the Kirby data. In order to transform these labels into each subject's DTI space, it was necessary to find a transform from the template to each subject's T1 and from T1 to DTI within each subject. For the template-to-T1 transform, the antsCorticalThickness.sh tool was used. This software first applied a bias correction using the N4 algorithm (Tustison et al., 2010). Next a registration based skull stripping was performed to provide a cerebrum mask of the T1 image. This was followed by a final cerebrum-only registration to the template. These transforms were composed with the T1-to-DTI transforms, providing a single transform that was used to warp the the AAL labels into DTI space using nearest neighbor interpolation. Labels of structures outside of the cerebrum were removed. Many AAL labels include both gray and white matter, here the labels were masked to only include voxels that were identified as cortical gray matter by the DKT31 labels described in the previous section. The AAL labels for deep gray structures (e.g., thalamus) were not masked but used in their entirety. Both label sets are illustrated in Figure 1, while the entire processing scheme is illustrated in Figure 2. The availability of the processing scripts is intended to provide a framework that allows for convenient exploration of alternate anatomical labels, such as the anatomical parcellations that may be obtained via FreeSurfer (http://surfer.nmr.mgh.harvard.edu) or the UCLA Multimodal Connectivity Package (http://ccn.ucla.edu/wiki/index.php/UCLA_Multimodal_Connectivity_Package), both of which have been used in previous graph-theory based examinations of structural connectivity based on diffusion-weighted imaging.

### 2.3. Diffusion data preprocessing

The Camino toolkit (Cook et al., 2006) was used to calculate diffusion tensor images via a weighted linear fitting (Basser et al., 1994; Salvador et al., 2005), and was used for subsequent deterministic tractography. The brain masks defined in T1 space were warped into DTI space and used to prevent tracking outside the brain. Fractional anisotropy (FA) images were calculated and a tractography seed-map was created to include all voxels in the cerebrum with an FA of at least 0.2.

One of the primary differences among the various approaches to deterministic tractography is the algorithm used to determine the direction that a streamline should proceed from a given point. Here we examine four different approaches:

1. Fiber Assignment by Continuous Tracking (FACT) - The primary direction of diffusion (PDD) is followed until the streamline enters a new voxel (Xue et al., 1999).
2. Euler—The PDD is followed for a constant step size (Basser et al., 2000).
3. Fourth-order Runge-Kutta (RK4)—The direction of the step is determined by taking and averaging a weighted series of partial steps (Basser et al., 2000).
4. Tensor Deflection (TEND)—The local fiber trajectory is a function of the previous direction and the local diffusion tensor (Lazar et al., 2003).

Shared parameters used in the fiber tracking were held constant as follows

1. Streamlines were terminated if curvature of more than 90° over 5 steps was detected.
2. Streamlines were terminated if an FA value of less of 0.2 was encountered.
3. A step size of 0.5 mm was used.
4. Linear interpolation of the primary direction of diffusion was used for Euler and RK4.

**Figure 3:**
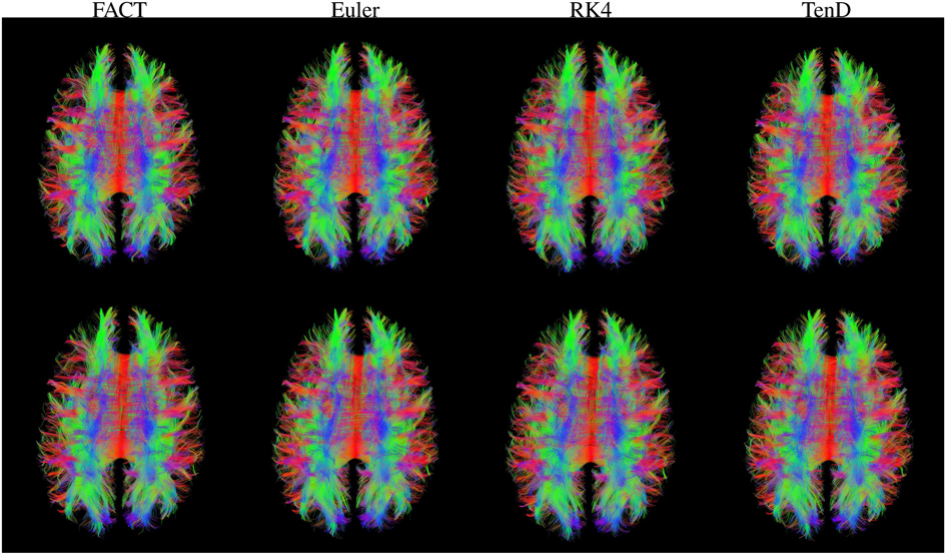
**Fiber tracts generated using each method are illustrated for both time points in a single subject**. For visualization, tract sets are sampled to display 5% of the tracts at 25% opacity. Tract points are colored to illustrate local streamline direction.

Figure 3 illustrates the fiber tracts for all methods for a single subject. The script used to generate these streamlines, deterministic_mmrr21.pl, is available as part of the git repository that contains all of the processing scripts for the work presented here (https://github.com/jeffduda/StructConnRepro). Relatively small changes to this script would allows users to explore additional deterministic tractograpy methods as well as probabilistic methods which are also available in the Camino toolkit.

### 2.4. Graph generation

While the nodes of a graph were defined using anatomical labels, the edges of the graph were defined by using fiber tractography to identify white matter pathways that connect brain regions. For a given set of streamlines, the connmat tool provided by the Camino toolkit was used to generate a connectivity matrix that records how many streamlines connect each pair of target regions. This program starts at the seed point for a streamline and proceeds outward in each direction to determines the two target regions encountered. Only streamlines that connect two unique regions are retained and a given streamline may be only be counted as connecting a single pair of target regions. Fiber tractography does not provide a measure of directionality (i.e., neither node can be considered a starting point or ending point) so the resulting matrices yield undirected graphs.

Graphs are often compared by first ensuring that they have the same density (Achard et al., 2006; Bassett et al., 2006), where density for an undirected graph is defined as:

$$D(G)=\frac{\left\|E(G)\right\|}{\left(\|N(G)\|(\|N(G)\|-1)\right)}$$

where *N*(*G*) is the set of all nodes in graph *G* and *E*(*G*) is the set of all edges in *G*. The number of nodes in the graph and the desired density determine the number of edges that the graph should contain. Edges of higher weights are given priority and lower weighted edges are removed to obtain the desired density level. The weights of the remaining edges are then set to 1 for a final binarized graph. This cumulative thresholding provides a normalized method for comparing network measures as it results in the comparison of graphs with an equal percentage of significant connections. Graphs are typically compared over a range of density levels. Here, we only directly compare measures obtained from graphs with an equal number of nodes and thus an equal number of edges after density thresholding.

### 2.5. Network metrics

A large number of graph metrics are available for quantifying properties of binary, undirected networks (Rubinov and Sporns, 2010). Here we examine a number that are common in current literature: largest connected component size (Bassett et al., 2011), assortativity (Newman, 2006; Bassett et al., 2008), clustering coefficient (Watts and Strogatz, 1998), characteristic path length (Watts and Strogatz, 1998), global and local efficiency (Latora and Marchiori, 2001), and rich club coefficient (Collin et al., 2013). An ITK module named Petiole (https://github.com/jeffduda/Petiole) was created to calculate these network measures from 2D connectivity matrices. This module incorporates and extends an existing implementation of a graph class (Tustison et al., 2008) and provides ITK functions for a variety of graph metrics while using the Matlab-based Brain Connectivity Toolkit (Rubinov and Sporns, 2010) for algorithmic guidance. While many of these metrics include implementations for weighted graphs and/or directed graphs, here we focus on their application to unweighted, undirected graphs. Summaries and equations for these metrics are provided here:

#### 2.5.1. Size of largest connected component

A connected component of a graph is a subset of the graph, *G*_*i*_, where there exists a path between all pairs of nodes and for which no path exist to additional nodes in *G*. The largest connected component is the *G*_*i*_ with the greatest number of nodes, ‖*N*(*G_i_*‖. This measure relates to the global level of connectivity within a subject's brain network (Bassett et al., 2011).

#### 2.5.2. Assortativity

The degree of a node is the number of neighboring nodes that it connects to (i.e., shares an edge with). Assortativity measures how preferentially nodes of similar degree connect to one another (Newman, 2006) and is defined as:

$$A=\frac{\frac{1}{E}\sum_{i}j_{i}k_{i}-{\left[\frac{1}{E}\sum_{i}\frac{1}{2}(j_{i}+k_{i})\right]}^{2}}{\frac{1}{E}\sum_{i}\frac{1}{2}(j_{i}^{2}+k_{i}^{2})-{\left[\frac{1}{E}\sum_{i}\frac{1}{2}(j_{i}+k_{i})\right]}^{2}}$$

where *j_i_, k_i_* are the degrees of the nodes connected by edge *i* and *E* = ‖*E*(*G*)‖. High assortativity suggests higher network resilience, making a network less vulnerable to attack (Newman, 2002).

#### 2.5.3. Clustering coefficient

This measure quantifies how likely is that two nodes with a common neighbor are connected to one another (Watts and Strogatz, 1998). Here we calculate the clustering coefficient at each node and calculate the mean over all nodes in the network for our final network summary measure. The clustering coefficient at node *i* is given by:

$$C_{i}=\frac{e_{i}}{\left\|K_{i}\|(\|K_{i}\|-1\right)}$$

where *K_i_* is the set of all nodes that share an edge with *i* and *e_i_* is the set of all edges that connect nodes in *K_i_*.

#### 2.5.4. Characteristic path length

The path length, *L*_*ij*_, that connects two nodes, *i* and *j*, is defined as the minimum number of edges that must be traversed to travel from *i* to *j* (Dijkstra, 1959). The characteristic path length is the average path length over all possible pairs of connections in a graph. In an undirected graph this is:

$$L=\frac{1}{\left\|N(G)\|(\|N(G)\|-1\right)}\sum_{ij\in G,i\neq j}L_{ij}$$

This measure is only defined for fully connected graphs. Here, we apply the density thresholding first and then extract the largest connected component in order to calculate the characteristic path length.

#### 2.5.5. Global efficiency

This measure is related to the characteristic path length, in that it attempts to quantify the mean efficiency between any two nodes in the graph. Unlike the characteristic path length, this metric is defined for both connected and unconnected graphs (Latora and Marchiori, 2001).

$$F_{glob}=\frac{1}{\left\|N(G)\|(\|N(G)\|-1\right)}\sum_{i\neq j\in G}1/L_{ij}$$

#### 2.5.6. Local efficiency

This metric relates to fault tolerance and examines efficiency between neighbors on a node *i*, if that node were removed from the graph (Latora and Marchiori, 2001).

$$F_{loc}=\frac{1}{\left\|N(G)\right\|}\sum_{i\in n}F(G_{i})$$

where *G_i_* is the subgraph of *G* that results from removing node *i*.

#### 2.5.7. Rich club coefficient

This measures quantifies how preferentially the high-degree nodes (i.e., rich nodes) in a graph connect to other high-degree nodes (Colizza et al., 2006).

$$R(G,k)=\frac{\left\|E(G,k)\right\|}{\left\|N(G,k)\|(\|N(G,k)\|-1\right)}$$

where *N*(*G, k*) is the set of nodes of degree k or higher and *E*(*G, k*) is the set of edges connecting two nodes in *N*(*G, k*).

### 2.6. Graph curves

The metrics listed above are all applied to thresholded binary graphs. As discussed earlier, these binary graphs result from thresholding at a constant density. The metrics may then be treated as functional curves of metric vs. graph density. By doing this, we are able to compare binary graphs in a way that incorporates the continuous structure of the original connectivity matrices. The rich club coefficient however is dependent upon two parameters, the graph density and *k*, the degree threshold used to determined what constitutes a rich-node. For this metric we threshold at the highest density common to all graphs and explore how the value changes with *k*. For all other metrics, we examine their curves as a function of graph density.

### 2.7. Statistical analysis

Before examining how the graph metrics change with density it is necessary to examine the maximum density of the graphs to determine the range over which graphs may be compared. Additionally, it is interesting to examine the topological similarity in the thresholded graphs. This is done using the dice coefficient which measures similarity between two graphs as:

$$Dice(x,y)=\frac{2\|E(x)\cap E(y)\|}{\|E(x)\|+\|E(y)|\;}$$

where edges are considered equal if they connect the same two nodes. This is equivalent to treating each connectivity matrix as a binarized 2D image and using the Dice metric to measure overlap. The mean intra- and inter-subject topological similarity was computed over a range of densities for each combination of tracking algorithm and anatomical label sets. This allows us to examine the reproducibility of within-subject topography compared to between subject topography. This metric is limited to lie in the range [0, 1] and can be interpreted as a measure of degree of overlap between graphs. This provides a stricter metric than measuring overlap between sets of nodes as complete node-overlap is a necessary but incomplete condition for complete edge-overlap.

Graph curves are used to examine the reproducibility of the graph metrics as a function of an independent parameter, typically graph density. At each point along the curve, reproducibility of the metric is quantified using the ICC:

$$ICC=\frac{\sigma_{bs}^{2}}{\sigma_{bs}^{2}+\sigma_{ws}^{2}}$$

where σ^2^_*bs*_ is the between-subject variance and σ^2^_*ws*_ is the within subject variance. The “ICC” package for R is used for this calculation. The ICC is plotted along with the mean graph metrics for each combination of algorithm and label set. At points where little to no variance exists in a graph metric, the ICC is not calculated as it becomes unstable under those conditions. The following guidelines may be used to interpret ICC values: ICC < 0.2 “poor agreement”; 0.21–0.40 “fair agreement”; 0.41–0.60 moderate agreement; 0.61–0.80 “strong agreement”; ICC > 0.8 “near perfect agreement” (Telesford et al., 2010; Montgomery et al., 2002). Dashed lines indicating the boundaries of these categories have been included on all ICC plots to aid interpretation.

To identify group differences that result from the fiber tracking algorithm we incorporated methods from functional data analysis (FDA), which treats each curve as a function. For each group, the set of all curves were averaged to create a single mean curve. While there are a variety of methods for computing the difference between two curves, here we chose the simplest method, the non-parametric permutation test. Each mean curve was treated as a function and the area between the group mean curves was found. Individual group assignments were then permuted using random sampling without replacement and then used to calculate mean curves. The area between the random-group mean curves was calculated. This was performed iteratively (*i* = 10000). We recorded *x*, the number of times the area between the mean curves from the randomly assigned groups is larger than the area between the true group mean curves. The *p*-value for the true group difference is then defined as *x/i*. We report these differences for between-algorithm curves as they derive from graph of equal size, but do compare curves that derive from different anatomical label sets.

## 3. Results

### 3.1. Network density

**Figure 4:**
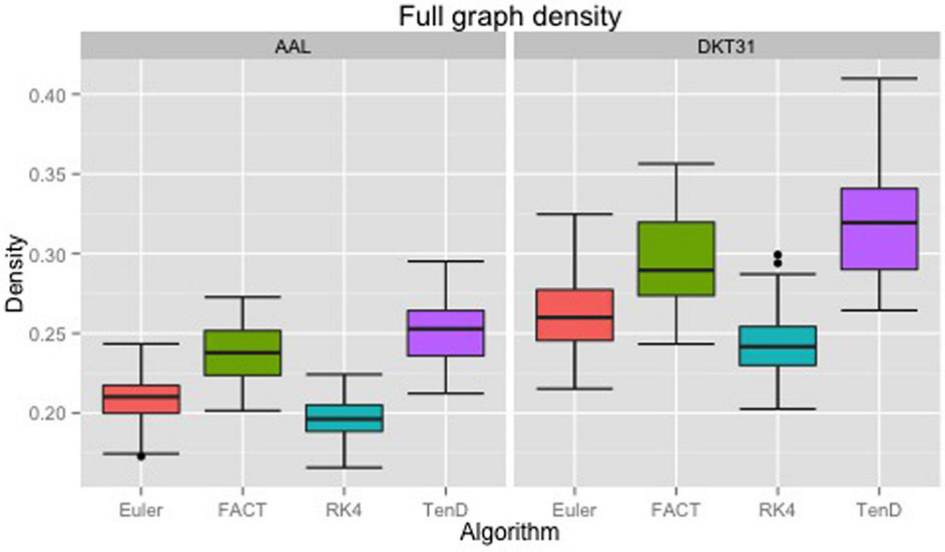
**Boxplots illustrating the density values for unthresholded connectivity matrices for all subjects and all time points, grouped by fiber tracking algorithm (Euler, FACT, RK4, TEND) and anatomical label set (AAL, DKT31)**. Black dots indicate data points whose distance from the hinge is more than 1.5 ^*^ inter quantile range.

Maximal densities for connectivity matrices across all tracking algorithms ranged from 0.17 to 0.30 for the AAL labels and from 0.20 to 0.41 for DKT31. Maximal densities in the DTK31 data was generally higher than in the AAL as illustrated in Figure 4. Both label sets had the same lowest-to-highest ordering of mean maximal density within algorithms: RK4 < Euler < FACT < TEND.

### 3.2. Network topology

**Figure 5:**
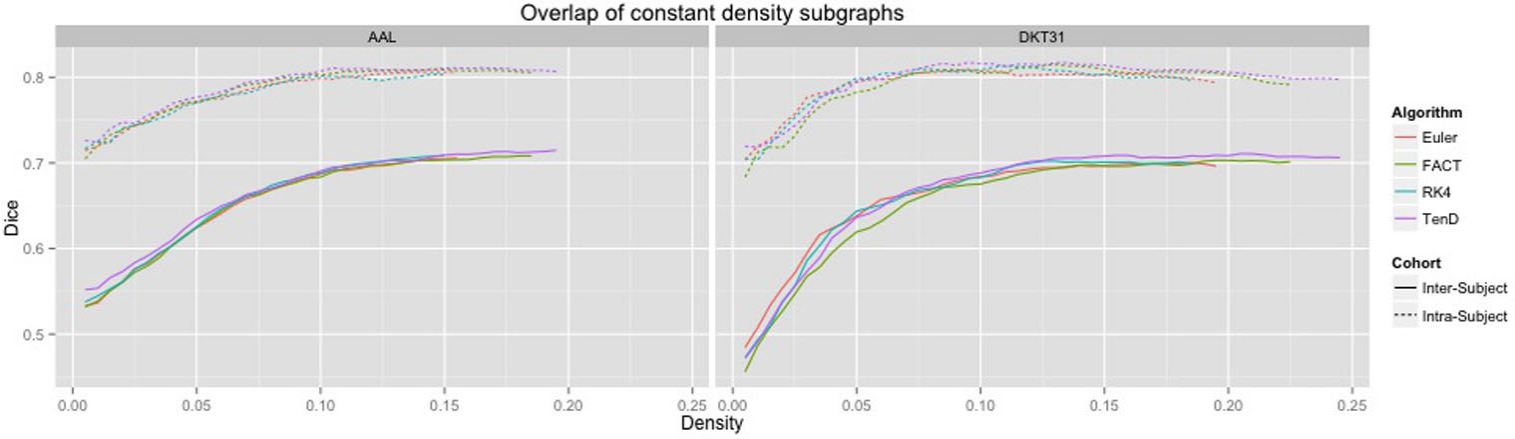
**Connectivity matrices were thresholded over a range of density values**. At each density level, consistency of network topography was estimated by calculating the mean dice overlap for both intra subject and inter subject pairs.

Dice coefficients for intra-subject similarity ranged from 0.70 to 0.81 for the AAL labels and from 0.59 to 0.82 for the DTK31 labels. Inter-subject similarity ranged from 0.51 to 0.71 for AAL labels and from 0.32 to 0.71 for the DTK31 labels. For all algorithm-label pairings, intra-subject overlap was greater than inter-subject overlap across the range of densities as illustrated in Figure 5. Permutation testing of intra-subject dice vs. density curves did not reveal any significant differences between algorithms for either label set. However, a number of differences were found in the inter-subject comparisons. The resulting *p*-values are listed in Table 1.

**Table 1:** **Functional data analysis is used along with permutation testing to look for differences in dice overlap measures between graphs generated from different fiber tracking algorithms**.

	**Intra subject**				**Inter subject**			
Euler		0.9555	0.9864	0.6549		0.7770	0.2675	0.0351^*^
FACT	0.4186		0.7970	0.9524	0.0002^*^		0.2586	0.0355^*^
RK4	0.8780	0.6179		0.5358	0.0632	0.0014^*^		0.1335
TEND	0.3952	0.6655	0.7564		0.0001^*^	0.0003^*^	0.0838	
	Euler	FACT	RK4	TEND	Euler	FACT	RK4	TEND

Upper triangular p-values are for the AAL labels, while lower triangular are for the DTK31 label set (

* *indicates significance at *p* = *0.05*)*.

### 3.3. Network summary measures over graph density

**Figure 6:**
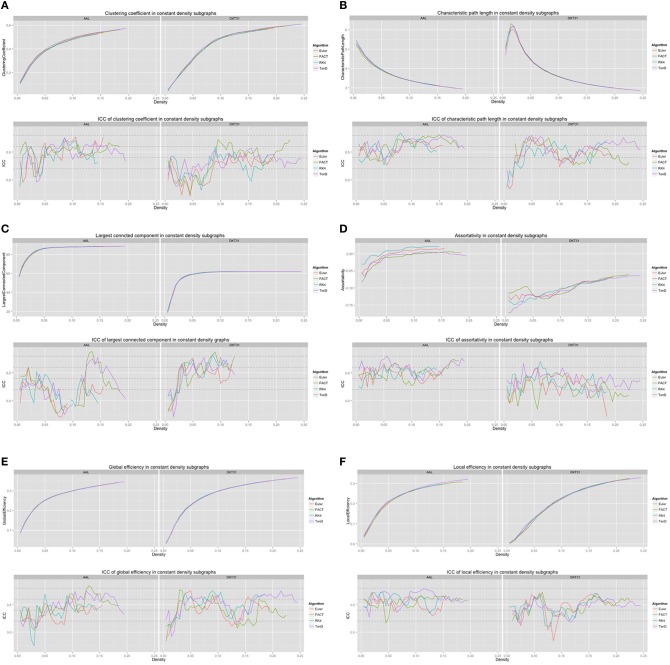
**Graph metric vs. graph density plots along with corresponding ICC plots for (A) mean clustering coefficient (B) characteristic path length (C) largest connected component size (D) assortativity (E) global efficiency, and (F) local efficiency**.

For each combination of tracking and label set, the mean curves that were calculated to examine how the metrics change as a function of graph density are illustrated in Figure 6 along with the ICC curves that quantify reproducibility. In some cases, ICC values may not be calculate due to insufficient variation in the metric. Only the characteristic path length curves exhibit a different shape between label sets, and only at low density values. This is likely a results of the smaller number of regions in DKT31 label set. Clustering coefficient, and global and local efficiency exhibit the most similarity across label sets. Comparing within metric and within label set, the fiber tracking algorithms appear consistent as far as shape. Functional data analysis, along with permutation testing does reveal a number of significant differences between graph curves however, as listed in Table 2. No significant differences were found between tracking algorithms using the DKT31 labels. Within the AAL labels, significant differences were found between RK4 and TEND for four of the six metrics examined.

**Table 2:** **Functional data analysis is used along with permutation testing to look for pair-wise differences in graph-metric vs. graph-density curves that result from different fiber tracking algorithms and label sets**.

	**Clustering coefficient**				**Characteristic path length**			
Euler		0.2164	0.5296	0.0346^*^		0.2786	0.2389	0.4728
FACT	0.6962		0.0246^*^	0.2822	0.9982		0.0145^*^	0.1235
RK4	0.9927	0.7958		0.0049^*^	0.8465	0.9199		0.3031
TEND	0.1327	0.8858	0.2061		0.4854	0.6459	0.8234	
	**Connected component size**				**Assortativity**			
Euler		0.3471	0.9324	0.7556		0.3680	0.3294	0.4651
FACT	0.9447		0.0468^*^	0.3025	0.6361		0.0270^*^	0.8877
RK4	0.9998	0.7610		0.4748	0.9326	0.3250		0.0666
TEND	0.7912	0.8336	0.7269		0.8272	0.4021	0.9895	
	**Global efficiency**				**Local efficiency**			
Euler		0.8617	0.9861	0.7272		0.4579	0.9227	0.6065
FACT	0.8677		0.6882	0.7667	0.8794		0.2486	0.1230
RK4	0.6295	0.9415		0.8677	0.9745	0.4413		0.7557
TEND	0.9977	0.9633	0.7438		0.8752	0.7987	0.4775	
	Euler	FACT	RK4	TEND	Euler	FACT	RK4	TEND

Only the first time-point for each subject is used. For each metric, the upper-triangular values are p-values for the AAL labels while the lower-triangular values were generated with the DKT31 label set (

* *indicates significance at *p* = *0.05*)*.

### 3.4. Rich club coefficient over node-degree

**Figure 7:**
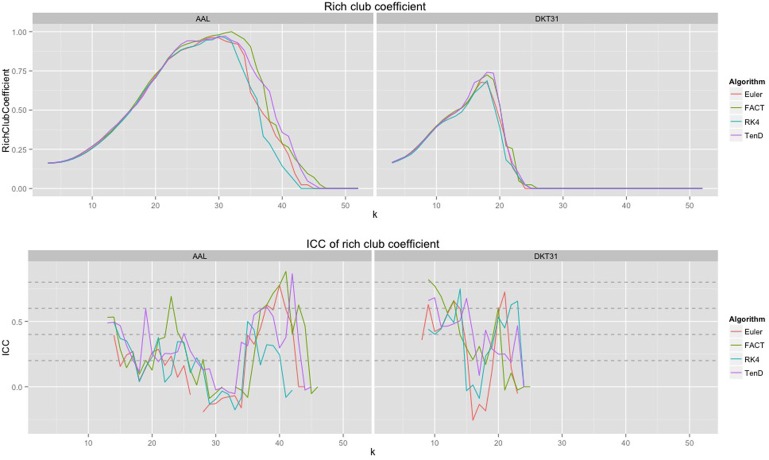
**Rich club coefficient was examined over a range of levels, k, and a constant graph density of 0.15**.

Because the rich club coefficient requires the selection of multiple parameters, we chose to examine how this metric changes as a function of *k*, the node degree that determines what is considered a “rich” node. The plots for the mean graph curves and ICC coefficients are illustrated in Figure 7. The results are similar to the examinations over graph density in that the same shape appears for both label sets, but with a scaling difference and the tracking algorithms have similar shapes but within the AAL networks, differences were found in the RK4-FACT (*p* = 0.0338) and RK4-TEND (*p* = 0.0252) comparisons. The *p*-values for all comparisons are listed in Table 3.

**Table 3:** **Functional data analysis is used along with permutation testing to look for differences in rich club coefficients generated from different fiber tracking algorithms**.

Euler		0.3359	0.6830	0.2064
FACT	0.8944		0.0338^*^	0.8537
RK4	0.9282	0.7219		0.0252^*^
TEND	0.7548	0.8800	0.3360	
	Euler	FACT	RK4	TEND

Upper triangular p-values are for the AAL labels, while lower triangular are for the DTK31 label set (

* *indicates significance at *p* = *0.05*)*.

## 4. Discussion

### 4.1. Network topology

Although a number of studies have examined the reproducibility of graph metrics on structural brain networks derived from DTI-based fiber tractography, there are no known papers that focus on the selection of deterministic tracking algorithm. To facilitate later examination of graph metrics as a function of graph density, we first examined the reliability of identifying subgraphs by thresholding. Using the dice coefficient as a measure of overlap we demonstrated that the intra subject agreement was much higher than the inter subject agreement across all tracking algorithms and label sets. No significant differences were found for intra-subject comparisons. The inter-subject comparisons indicate that the TEND method most consistently identifies similar subgraphs at a given density. However, further analysis of additional tracking parameters is necessary to determine the full set of conditions under which this result holds.

### 4.2. Network summary measures over graph density

Global and local efficiency are the most robust to choice of fiber tracking algorithm, and have high levels of reproducibility across density levels. Assortativity and characteristic path length are highly reproducible across density levels, but are sensitive to choice of fiber tracking algorithm. In general, the portions of the graph curves at low density value and less reproducible than the segments at high density. For many metrics, the graph curves strongly converge at high density values suggesting that examining the metrics at those densities may be of little use. Examining both the mean graph curves and ICC plots may provide guidance for the range of densities that should be looked at in a group comparison study.

### 4.3. Rich club coefficient over node-degree

The examination of rich club coefficient as a function of degree-level demonstrates the use of graph curves over a parameter other than graph density. Consistency appears to have a somewhat inverse relationship to the coefficient as a function of node degree level. This is a result of the fact that the rich club coefficient values converge at high and low densities. Here, all graphs were thresholded at the maximum density achievable by all graphs. Because network size is held constant here, the average node degree would drop with lowered density, and additional work is required to more understand the relationship between graph-density and degree-level that would provide the most reproducible results.

### 4.4. Limitations and future directions

The are a number of methodological limitations to the work presented here. We limited the fiber tracking to deterministic methods and used constant shared parameters for these methods. The influence of these parameters on individual tracking algorithms and the resulting graph metrics demands further exploration. In the choice of anatomical label sets, we limited the analysis to a set of manually defined labels, and a often used set of template-based labels. In each case we used the labels “as-is” without upsampling to a higher number of regions. This may reduce the reproducibility of the networks, but provides more interpretable results if one wishes to examine individual connections or a subset of connections (e.g., default mode network) since the labels have well defined anatomical associations.

The use of a data with relatively low angular resolution and the use of the diffusion tensor model are both limiting factors in the work presented here. To some degree, the choice of diffusion model is limited by the data used here. However, these limitations are representative of a great deal of existing data sets. Thus, the work presented here provides insight into the utility of using this data to examine network-wide structural connectivity properties. Additionally this work provides a baseline analysis. This allows methods using more sophisticated techniques, such as diffusion spectrum imaging and it's associated models, to demonstrate the added value of those methods. Without this baseline, the added benefit of these more complex techniques is less clear due to a lack of sufficient context.

An additional limitation of this work is the use of streamline count matrices as the basis for thresholding to create constant density graphs. Multiple options exist for normalizing the streamline count matrices using the volumes of the target cortical regions and/or the average length of the streamlines the connect two regions. The volume based normalization may accommodate the differences that are seen between graph curves that were generated using the different anatomical labels. However, the focus here was on the influence of the fiber tracking and no direct comparisons were made between graph curves generated from the different label sets. A number of additional options exist for creating a weighted connectivity matrix including the average FA of fibers that connect two regions. Since the data set examined also includes magnetization transfer data, the average magnetization transfer ratio along streamlines could potentially be useful as it directly related to myelin content in white matter. These issues were beyond the scope of the current study but would make for an intriguing extension of the current work.

The selection of graph metrics for analysis is another limitation of the study. An exhaustive examination of all possible graph metrics was not feasible so metrics that have been studied previously were chosen to give additional context to existing work. Many of the metrics examined have alternate formulations for weighted graphs. Here, only unweighted graph metrics were examined as they are prevalent in current literature. The creation of a testing framework that relies upon a public data set and open-source code was intended to facilitate the further exploration of the issues listed here.

### 4.5. Conclusion

This study evaluate the reproducibility of graph summary metrics in structural brain networks derived from DTI based deterministic fiber tractography. Four different fiber tracking algorithms were examined along with two different anatomical label set. A number of graph metrics were examined by creating graph curves that capture how a metric changes over a parameter such as graph density. ICC plots were used to evaluate the reproducibility of the metrics and FDA was used to identify significant differences between graph curves generated using different fiber tracking algorithms. While differences between the tracking algorithms were not drastic, they were significant in many cases, suggesting that future studies should give careful consideration to the choice of fiber tracking algorithm based upon the graph metric that will be analyzed.

### 4.6. Data sharing

Free, publicly-available data and software was used throughout. The scripts used to generate the data and figures are available at: https://github.com/jeffduda/StructConnRepro. This repository contains the configuration file that, when added to ITK, will download and compile Petiole which builds the executables that were used to generate the graph metrics examined in this study. The template with labels is available at http://figshare.com/articles/Kirby_multivariate_template/852989, the final segmentations used as the target regions for fiber tracking are available at http://figshare.com/articles/MMRR21_DTI_Targets/850369 to provide a convenient starting point for reproducing or extending the methods presented here.

### Conflict of interest statement

The authors declare that the research was conducted in the absence of any commercial or financial relationships that could be construed as a potential conflict of interest.
